# Tolerogenic dendritic cells in experimental autoimmune encephalomyelitis, specific tolerance induction?

**DOI:** 10.1186/1479-5876-10-S3-P27

**Published:** 2012-11-28

**Authors:** Carla Sellés-Moreno, Cristina Ramo-Tello, Laia Grau-López, Ricardo Pujol-Borrel, Eva M Martínez-Cáceres

**Affiliations:** 1 Immunobiology Laboratory For Research And Diagnostic Applications (LIRAD-BST), Badalona, Spain; 2 Multiple Sclerosis Unit, Dept. of Neurosciences, Germans Trias i Pujol University Hospital (HUGTiP) and Health Science Research Institute (IGTP), Badalona, Spain; 3 Dept. of Immunology, Hospital Vall d’Hebron, Barcelona, Spain; 4 Universitat Autònoma Barcelona, (UAB), Spain

## Background

Specific cell therapy with tolerogenic Dendritic Cells (tolDCs) loaded with autoantigens is a promising tool for the attenuation of pathogenic T cells in autoimmune diseases such as Multiple Sclerosis (MS).

The aim of this study was to analyse the in vitro effect of tolerogenic bone marrow derived DCs (BM-DCs) loaded with Myelin Oligodendrocyte Glycoprotein (MOG)40-55 peptide from C57BL/6 mice, on the specific proliferation of splenocytes of mice with EAE.

## Methods

Chronic EAE was induced in C57BL/6 mice by s.c. immunization with MOG40-55 emulsified in complete Freund's adjuvant. Pertussis Toxin was injected i.v. at days 0 and 2 post-immunization to increase blood-brain-barrier permeability. Clinical score was daily measured based on tail/leg paralysis.

TolBM-DCs were in vitro differenciated with GM-CSF in the presence of vitamin D3 (VD3) for 8 days.

On day 7, maturation was induced with LPS. Viability, efficiency of differentiation and phenotype of tolBM-DCs were evaluated. To assess antigen specificity, tolBM-DCs were pulsed with MOG40-55, after the maturation stimulus (day 7) for 18h and cocultured with syngeneic splenocytes from mice with established EAE.

## Results

TolBM-DCs displayed a semi-mature phenotype, exhibited by low levels of MHC class II and coestimulatory molecules (CD40, CD86) compared to control mature BM-DCs (mBM-DCs) differenciated in the absence of VD3. MOG40-55 loaded TolDCs showed to be poor stimulators of specific T cells of mice with EAE, compared to mBM-DCs.

## Conclusion

These results suggest that MOG-loaded TolDCs may be a powerful tool to induce specific T-cell hyporesponsiveness in mice with chronic MOG-induced EAE. Hence, treatment with tolDCs loaded with myelin peptides might be a potential therapy for EAE/MS.

